# Towards an adequate description of the dose‐response relationship in BNCT of glioblastoma multiforme

**DOI:** 10.1002/mp.17693

**Published:** 2025-02-22

**Authors:** Barbara Marcaccio, Marco Crepaldi, Ian Postuma, Erica Simeone, Claretta Guidi, Setareh Fatemi, Ricardo Luis Ramos, Valerio Vercesi, Cinzia Ferrari, Laura Cansolino, Elena Delgrosso, Riccardo Di Liberto, Daniele Dondi, Dhanalakshmi Vadivel, Yi‐Wei Chen, Fong‐In Chou, Jinn‐Jer Peir, Chuan‐Jen Wu, Hui‐Yu Tsai, Jia‐Cheng Lee, Agustina Mariana Portu, Ana Mailén Dattoli Viegas, Sara Josefina González, Silva Bortolussi

**Affiliations:** ^1^ Department of Physics University of Pavia Pavia Italy; ^2^ National Institute of Nuclear Physics (INFN) Unit of Pavia Pavia Italy; ^3^ Universidad Nacional de San Martín San Martín Buenos Aires Argentina; ^4^ Department of Biology and Biotechnology University of Pavia Pavia Italy; ^5^ Department of Clinical and Surgical Sciences, Integrated unit of experimental surgery, Advanced microsurgery and regenerative medicine University of Pavia Pavia Italy; ^6^ Foundation I.R.C.C.S. San Matteo Polyclinic Pavia Italy; ^7^ Department of Chemistry University of Pavia Pavia Italy; ^8^ Department of Heavy Particles and Radiation Oncology Taipei Veterans General Hospital Taipei Taiwan; ^9^ Nuclear Science and Technology Development Center National Tsing Hua University HsinChu Taiwan; ^10^ Institute of Nuclear Engineering and Science National Tsing Hua University HsinChu Taiwan; ^11^ Comisión Nacional de Energía Atómica (CNEA) Buenos Aires Argentina; ^12^ Consejo Nacional de Investigaciones Científicas y Técnicas Buenos Aires Argentina

**Keywords:** BNCT, cell survival curves, dosimetry, glioblastoma multiforme

## Abstract

**Background:**

Boron Neutron Capture Therapy (BNCT) is a binary radiotherapy based on the intravenous administration of a borated drug to the patient and the subsequent irradiation with a low‐energy neutron beam. The borated formulation accumulates in the tumor cells, and when neutrons interact with boron, a nuclear capture reaction occurs, releasing high‐linear energy transfer, short‐range particles that cause lethal damage to the cancer cells. Due to its selectivity, BNCT has the potential to treat aggressive brain tumors such as glioblastoma multiforme (GBM), minimizing the side effects. GBM is a brain neoplasia that poses significant treatment challenges due to its invasiveness and resistance to conventional treatments.

**Purpose:**

This work aims to find a suitable model for calculating the photon isoeffective dose for GBM, producing ad hoc radiobiological data to feed the model.

**Methods:**

To describe adequately the dose‐effect relation of BNCT for GBM, the following strategy has been applied

1. We studied the impact of choosing two different photon radiation types (x‐ or gamma‐ rays)
2. We assumed that the correct description of the photon‐equivalent dose is obtained with the photon isoeffective dose model. This model calculates the photon dose that equals the cell survival obtained with BNCT, taking into account synergism and sub‐lethal damage (SLD).
3. Survival curves as a function of the dose for the human GBM U87 cell line were constructed using the clonogenic assays for irradiation with photons (reference), neutron beam, and BNCT.
4. Survival curves were fitted with the modified linear quadratic model, using SLD repair times derived for U87. The radiobiological parameters were determined for the photon isoeffective dose model.
5. The model was applied to a clinical case that received BNCT in Taiwan. Treatment planning has been simulated using an accelerator‐based designed neutron beam following the real treatment process and parameters. The results were discussed and compared to the current method, which employs relative biological effectiveness (RBE) factors to obtain BNCT dosimetry in photon‐equivalent units.

**Results:**

The dose‐survival curves have been obtained with two different photon radiation sources as the reference with a thermal neutron beam and neutrons in the presence of boron. The fitted parameters have been obtained as the input for the photon isoeffective dose and the traditional RBE model. For the first time, the radiobiological parameters of a photon isoeffective dose model were produced for BNCT of GBM. Photon isoeffective dose value can differ up to 32% using gamma photons and low‐energy x‐rays. Photon isoeffective dose values are lower (17%) than the RBE model currently employed in clinical trials.

**Conclusion:**

The results highlight the impact of the reference radiation chosen for the isoeffective dose calculation and the importance of feeding the model with the appropriate radiobiological parameters.The dosimetry obtained with the new radiobiological data is consistent with the dose delivered in modern stereotactic radiotherapy, enabling tumor control predictions.

## 1 INTRODUCTION AND PURPOSE

Boron Neutron Capture Therapy (BNCT) is a highly selective radiotherapy based on the neutron capture reaction 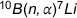. Boron‐10 (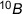) is administered to patients via compounds capable of loading the tumor with higher concentrations of the isotope compared to that obtained in healthy tissue. A low‐energy neutron irradiation produces the capture reactions inside the tumor cells, and the resulting alpha and 7‐lithium ions are highly ionizing particles, releasing all their energy in a track length of 5−9μm. Thus, it is possible to impair the vital functions of tumor cells while preserving healthy tissues, where a lower number of neutron captures occur. This treatment has been applied in several countries to treat tumors with limited or no response to traditional therapies. Neutrons have been obtained from nuclear reactors and, more recently, from proton accelerators coupled to Be or Li targets, capable of delivering neutron beams with suitable spectral characteristics for the therapy.^1^

In Italy, the ANTHEM project, funded by the Complementary National Plan (PNC) in the scheme of the National Recovery and Resilience Plan (PNRR), foresees the construction of a clinical center based on the proton accelerator Radiofrequency Quadrupole accelerator (RFQ) designed and built by the Italian National Institute of Nuclear Physics (INFN), at the National Laboratory of Legnaro.^2^ This accelerator, coupled with a Be target and a suitable beam shaping assembly, can deliver a neutron beam with the same therapeutic potential as the one of FIR‐1 beam in Finland, where hundreds of patients were treated.^3^, ^4^ ANTHEM has a special focus on the treatment of brain tumors, especially those that are currently orphan, such as glial tumors. Among these, glioblastoma multiforme (GBM) stands out for its malignancy, rapid progression, and resistance to conventional treatments, that is, irradiation and chemotherapy after surgery. This tumor is characterized by glial hypercellularity, atypical nuclei (nuclear pleomorphism, multinucleation, coarse nuclear chromatin), visible mitotic activity, and prominent vascular proliferation with endothelial hyperplasia, often so intense as to cause vascular obstruction. These factors cause the formation of abundant areas of tissue necrosis that, in turn, act as a hypoxic stimulus inducing angiogenesis. GBM is associated with deterioration of neuro‐cognitive functions,^5^, ^6^ reduced functional independence, and a progressive decrease in quality of life.^7^, ^8^ The current standard treatment for GBM is total surgical resection whenever possible, followed by radiotherapy combined with temozolomide‐based chemotherapy (TMZ). Despite this, most patients undergo very rapid tumor progression, with a survival time of about 14 months and a 5 year survival rate of less than 5%.^9^

BNCT has the potential to play a key role for this malignancy. In recent years, BNCT has been performed on patients with brain tumors with encouraging clinical outcomes.^10^, ^11^ The establishment of new BNCT centers not only drives advancements in beam technology for enhanced effectiveness but also requires improvements in dose calculation, in the comprehension of radiobiological dose‐effect relationships, and in the ability to predict the therapeutic outcome based on treatment planning simulations. The total absorbed dose in tissues due to BNCT is the consequence of the interaction of a mixed field of radiation, each component with different biological effectiveness. The complexity of this field makes it difficult to predict the therapeutic effect for a given absorbed dose. Therefore, it is necessary to translate the BNCT dose to a dose due to conventional photon radiotherapy, for which the dose‐effect relationship is well known.

Traditionally, the BNCT absorbed dose has been expressed in photon equivalent units (Gy (RBE)) by multiplying each of the four components, in Gy, by the corresponding relative biological effectiveness (RBE) or the compound biological effectiveness (CBE) for the different radiations.^12^ These factors are determined by radiobiological experiments for a given biological system and effect level (endpoint). The RBE is defined as the ratio of the doses required by two radiations to cause the same level of effect. In BNCT, the RBE of the neutron beam is usually obtained as the ratio of the photon dose and the neutron beam dose needed to cause a certain cell survival obtained by in vitro radiobiological experiment. The CBE is the RBE of the boron dose component: it is boron carrier‐dependent since boron biodistribution achieved with a specific drug can produce different cell damage for the same boron concentration.

In 2012, González and Santa Cruz demonstrated that the use of such constant factors results in artificially high tumor dose values, which fail to explain the clinical outcome of BNCT treatments compared to conventional photon radiotherapy.^13^ This study thus highlights that RBE‐weighted doses are not equivalent to photon doses. To obtain a more reliable equivalence with photons, the authors developed a new dosimetric model, which calculates the photon isoeffective dose, expressed in Gy (IsoE), defined as the photon dose that produces the same effect (for example, the probability of tumor control or the probability of cell survival) as a given combination of the four BNCT dose components. This model has been adopted in our studies about beam optimization and study of BNCT feasibility using the neutron beam obtained with the INFN accelerator.^4^ To feed the model, different types of radiobiological data can be used. In Pavia, we produce cell survival curves as a function of the dose by irradiation of in vitro models with a detailed calculation of the dose absorbed by the cell monolayers.^14^, ^15^

The dosimetry of the GBM patients has always been calculated using the radiobiological parameters of gliosarcoma (GSM). GSM contains a portion that matches the histological criteria for GBM and a mesenchymal component that can present a variety of morphologies originating from fibroblastic, cartilage, bone, smooth muscle, striated muscle, or adipose cells.^16^ GSM is considered a close sibling of GBM. Studies show that this tumor has a unique and distinct clinico‐pathological entity compared to GBM, and therefore, a precise classification is necessary for appropriate treatment planning.^17^

This work presents for the first time a suitable model for the calculation of photon isoeffective dose in GBM, producing ad‐hoc in vitro data. To this end, we irradiated cell cultures of human glioblastoma (U87 cell line), evaluating the radiobiological parameters for an accurate photon isoeffective dose model for this tumor. The work describes:

1. the production and the analysis of new radiobiological data using the U87 cell line;
2. the evaluation of impact in using different photon sources as the reference radiation;
3. the comparison of photon isoeffective dose obtained with different sets of radiobiological data in a GBM patient who received BNCT in Taiwan.

## 2 MATERIALS AND METHODS

The production of new radiobiological data required a set of irradiation experiments, supported by dosimetry calculations for different types of radiation. Dose‐survival curves are produced, for GBM, with the human cell line U‐87 GM, abbreviation for Uppsala 87 Malignant Glioma (U87). This line represents one of the main reference models for the study of human GBM in vitro. The cells were initially isolated from a 44 year‐old female glioma brain patient at the Uppsala University, Sweden, in 1966.^18^ Cell survival was evaluated under the effect of different types of radiation and for increasing dose. Adherent tumor cells at 60‐80% confluence were irradiated with neutrons, and with neutrons after administration of the borated molecule Boronophenylalanine (BPA) at the TRIGA Mark II research reactor, LENA laboratory, Pavia University, Italy. The reference curve is obtained by photon irradiation.

The calculation of the photon isoeffective dose aims to leverage the dose‐effect knowledge from clinical photon therapy to provide more effective BNCT treatments and predict the therapeutic outcomes. Therefore, it is imperative that the reference radiation accurately reflects the conditions of clinical radiotherapy. While typical radiobiological experiments often utilize x‐ray as the reference, some studies suggest that this may not provide a sufficiently representative setting.^19^ Moreover, one of the components of BNCT mixed field is made up by photons, mainly 2.2 gamma rays coming from neutron capture in hydrogen. As explained below, one of the assumptions for the development of the photon isoeffective dose model is that the radiobiological parameters of the reference radiations can be used to describe the photon component of BNCT. Thus, a reference radiation more similar to the 2.2 MeV gamma component is preferable. In this work, we addressed this issue by irradiating U87 cell cultures using two distinct photon sources: a low‐energy x‐ray irradiator (with an average energy of 60‐80 keV) and a Co‐60 source, whose energy spectrum is more similar to that of conventional radiotherapy. Viegas et al.^15^ showed the importance of calculating detailed dosimetry in radiobiological experiments. We demonstrated that assuming charged particles equilibrium (CPE) conditions leads to an incorrect interpretation of survival data, which then propagates to the patient dose calculation. For this work, the detailed dosimetry obtained by Monte Carlo calculation described in the cited article was extended to the photon component.

### 2.1 Neutron irradiation

Neutron irradiation for measuring cell survival was performed in the thermal column of the TRIGA Mark II reactor (Pavia). The facility provides a thermal neutron field with low epithermal and fast neutron components and low gamma contamination.^20^ For irradiation in presence of boron (BPA‐BNCT), cells were incubated 48 h before irradiation in T‐75 flasks in 10 mL of culture medium enriched by the BPA for 4 h. Subsequently, they were washed three times with phosphate buffered saline (PBS) and fresh culture medium was supplied, to study the effect of the boron absorbed by the cells.

The dose component due to neutron capture in boron depends on 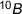 concentration internalized by the cells. For this reason, a flask of cells treated as those exposed to the neutron beam was prepared for boron concentration measurement by neutron autoradiography.^21^ Dose is calculated as described in ref. [15].

The dose was escalated by varying the reactor power (7.5 kW, 30 kW, 100 kW and 250 kW), and the irradiation time was fixed to 10 min, to minimize the time the cells stay outside the incubator. The cell survival was evaluated via the clonogenic assay.^14^, ^15^

For survival curves without BPA administration (*beam‐only*), cells were prepared as described above and again the clonogenic test was performed to evaluate the percentage of cells that survived the irradiation. Cultures of U‐87 cells administered with BPA at the same conditions as mentioned above were prepared to verify that it does not affect the survival. Results showed that the survival of BPA‐supplemented cells is not different that the control one, demonstrating the fact that BPA alone is not toxic at these concentrations.

### 2.2 Photon irradiation

For photon irradiation, cells were prepared as for neutron irradiation, using T‐25 flasks.

#### X‐ray irradiation

**FIGURE 1:**
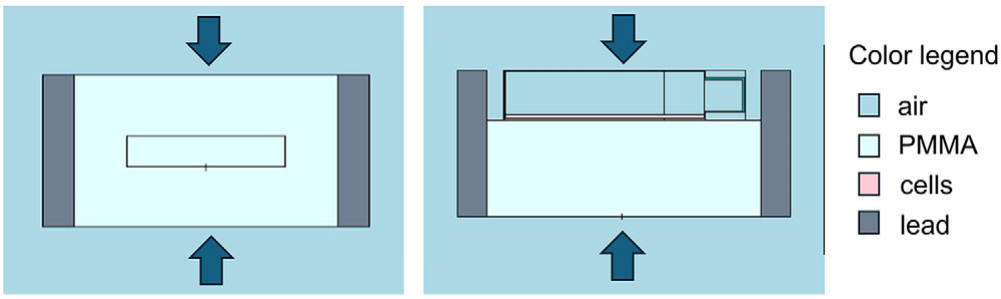
Model of the x‐ray irradiator set‐up. The sample into the tissue‐equivalent material (PMMA) is irradiated from the top and the bottom by two x‐ray beam of average energy 60–80 keV (arrows). Left: the central rectangle represents the calibration position where the detector is irradiated in CPE conditions. Right: The upper part of the phantom is removed and the flask is irradiated in the upper part of the drawer. CPE, charged particles equilibrium; PMMA, polymethylmethacrylate.

The first set of photon irradiation took place at the San Matteo Polyclinic in Pavia, using a ${\text{Best}}^{TM}\text{Theratronics}$ irradiator (Raycell®Mk2 X‐ray blood irradiator), commonly used to sterilize blood bags for transfusions. This device consists of a lead‐shielded chamber containing two opposing 160 kW x‐ray sources (average x‐ray energy 60‐80 keV). The facility has a removable drawer housing a stand in which samples are placed and irradiated. The drawer contains a polymethylmethacrylate (PMMA) tissue‐equivalent phantom with housings for small samples irradiation and for the calibration detector. The calibration of the set‐up had been obtained with an ionization chamber positioned in the middle of the PMMA phantom, in CPE conditions. However, the cell cultures had to be positioned on the top of the phantom, as the detector housing was too small to host the flasks. This could impair the CPE conditions and the nominal dose, as the position of the flask was closer to one of the two x‐ray beams and without phantom on the top. To evaluate the impact of this, a simplified Monte Carlo model was built with the code MCNP6.1, representing the drawer with lead walls, the PMMA phantom, the housing for the detector, the flask and the two opposite x‐ray beams. The cells were modeled as a 10 μm thick layer of soft tissue (Figure 1). Two simulations were carried out: one calculating KERMA (F6‐type tally) and dose (+F8‐type tally for electrons divided by mass) in the detector position, and the other calculating KERMA and dose in the cell monolayer. The ratio of the values obtained at the detector position and in the cells allowed a proper correction of the nominal dose values. The statistical error associated to the Monte Carlo results was below 1%.

The results showed that: (1) KERMA and dose have the same value in the detector position, as expected; (2) in the cell layer, KERMA and dose differ by 9%; (3) the total correction taking into account the lack of CPE and the distance from the calibration position is 16%. This correction was thus applied to the nominal dose when building the dose‐survival curve.

#### Cobalt‐60 photon irradiation

The second set of photon irradiation was carried out at the LENA laboratory, at the Co‐60 facility. Five flasks containing the U87 cell line in adhesion were irradiated simultaneously within a dedicated well surrounded by 13 cobalt rods. The 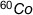 decays into 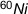 with a half‐life of 5.3 years, emitting two photons of energy 1.1732 MeV and 1.3325 MeV. The dose rate was about 1 Gy/min, determined by previous calibration with an ionization chamber and alanine dosimeters.

T‐25 flasks were inserted vertically into the photon field and extracted after different time intervals to deliver five different dose values, assuming CPE conditions in the cells. To guarantee the equilibrium, flasks were completely filled with culture medium and a dummy water‐filled flask was used to occupy the empty space into the holder. The assumption of CPE was studied in detail with a dedicated Monte Carlo simulation using the radiation transport code MCNP6.1 reproducing the irradiation set‐up with the flask inserted, and the photon source distribution.

**FIGURE 2:**
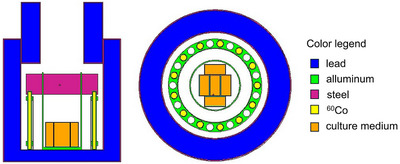
Left: vertical section of the facility for Co‐60 irradiation, right: horizontal section of the facility.

Figure 2 shows a vertical section of the set‐up, where the flasks are represented in orange, and a horizontal view where the rods emitting photons are represented in yellow.

As before, dose was calculated in the 10 μm cell layer, both assuming CPE (F6‐type tally) and with detailed transport of the secondary electrons (F8*‐type tally divided by cell mass). The statistical error associated to the Monte Carlo results was below 1%. In this case, the two values obtained were compatible, confirming the CPE condition and allowing for the use of nominal dose absorbed by cells.

### 2.3 Radiobiological survival curves

The cell survival as a function of the dose were fitted with the model described extensively in ref. [13]. The equations are

(1) $$S_{R}=exp\left[-\left(\alpha_{R}D+\beta_{R}G_{R}\left(\theta \right)D^{2}\right)\right],$$

and

(2) SBNCT=expβγβn−βγβnαBDB+αnDn+αγDγ+GBβBDB2+GnβnDn2+GγβγDγ2+2GBnβBβnDBDn+2GBγβBβγDBDγ+2GγβγβnDγDn,

where $\alpha_{R}$ and $\beta_{R}$ are the radiobiological parameters of the reference photon radiation *R*, $\alpha_{i}$ and $\beta_{i}$ with *B* = boron, *n* = neutrons and γ = photons, the radiobiological parameters of the different radiation components of BNCT. The model takes into account the first‐order repair of sub‐lethal damage (SLD) employing the Lea‐Catcheside temporal *G*‐factors and the synergism between the different radiation components through the quadratic mixed dose terms.

Mathematically, it is possible to describe the factor G with the dual kinetics of slow $\left(t_{0s}\right)$ and fast $\left(t_{0f}\right)$ repair as follows^22^

(3) $$G_{R}\left(\theta ,t_{0f},t_{0s}\right)=a_{Rf}G\left(\theta ,t_{0f}\right)+a_{Rs}G\left(\theta ,t_{0s}\right),$$

where $a_{Rf}$ and $a_{Rs}$ are the proportions of sublesions repaired by the fast and slow kinetics for radiation *R* (with $a_{Rf}+a_{Rs}=1$), and

(4) $$G\left(\theta ,t_{0}\right)=\frac{2t_{0}}{\theta }\left(1-\frac{t_{0}}{\theta }\left(1-e^{-\frac{t_{0}}{\theta }}\right)\right),$$

where $t_{0}$ is the fast or the slow characteristic repair time.

The Equation 1 was used to fit the experimental data obtained from photon irradiation, and to achieve the radiobiological coefficients $\alpha_{R}$ and $\beta_{R}$ specific to the U87 cell line. The alpha and beta parameters of the gamma component of the BNCT dose can be assumed to be equal to those of the reference radiation (as said before, this is especially true when the reference radiation source is Co‐60). In this case, the following equivalences hold

(5) $$\begin{array}{c} \alpha_{R}=\alpha_{\gamma },\\ \beta_{R}=\beta_{\gamma }. \end{array}$$

Once the radiobiological coefficients for the gamma component had been obtained, the cell survival model described in Equation 2 was used to calculate the remaining radiobiological parameters. The G factors for each dose point remain the same when the dose increases because irradiation is performed at a fixed time. The kinetic repair time was obtained using experimental data published in ref. [23], consisting in the normalized number of foci/nuclei at different time intervals after 2 Gy/min photon irradiation for U87 cell line. For a quantitative description of the progress of DNA damage reduction, the authors of the cited paper proposed a mono‐exponential fit. However, in this work we showed that a bi‐exponential equation (Equation 6) with a fast ($t_{0f}$) and a slow ($t_{0s}$) component better fits the data, c being the proportion that repairs with the fast kinetics.

**FIGURE 3:**
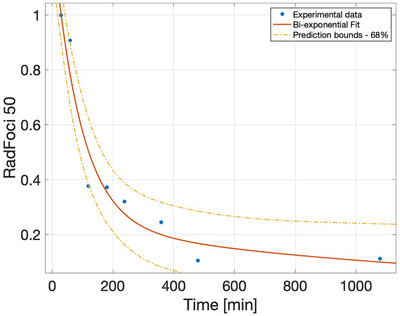
Kinetic repair time data (dots) from^23^ and bi‐exponential fit (solid line). The dash‐dot lines represent the upper and lower bounds of the 68% confidence interval. The dash‐dot orange lines represent the upper and lower bounds of the 68% confidence interval. The fit has a mean squared error (MSE) of 0.86.

Figure 3 shows the fitted curve.

(6) $$I\left(t\right)=ce^{-(1/t_{0f})t}+\left(1-c\right)e^{-(1/t_{0s})t}.$$

As a result of the fit, we obtained the two characteristic repair times and the percentage of SLD repair for low Linear Energy Transfer (LET) radiation. The values of the percentage of SLD repair for high LET radiation were taken from.^22^ Based on what is described in ref. [22], the repair times for high LET radiation are assumed to be the same as for the low LET radiation.

Table 1 summarizes the results obtained in this work for the U87 cell line along with the relative percentage of cells repaired by fast and slow kinetics for both photon and high‐LET radiation. Uncertainties correspond to a 68% confidence level.

**TABLE 1:** Values of the characteristic repair times and the corresponding percentage of SLD repair.

		SLD repair [%]	
	Characteristic repair times	Low LET	High LET^b^
$t_{0f}$	91 ± 45 min	0.77 ± 0.54^a^	0.2
$t_{0s}$	1238 ± 1237 min	0.23 ± 0.54	0.8

*Note*: Uncertainties correspond to a 68% confidence level.

^a^ Constant c in Equation 6.

^b^ Data taken from.^22^

Abbreviation: LET, linear energy transfer; SLD, sub‐lethal damage.

As mentioned above, the photon isoeffective dose $D_{IsoE}$ is the photon dose that produces the same effect as a given combination of the four BNCT dose components. Thus $D_{IsoE}$ is obtained by solving:

(7) $$S_{R}\left(D\right)=S_{BNCT}\left(D_{B},D_{n},D_{\gamma }\right).$$

So the final expression for the photon isoeffective dose is:

(8) $$D_{IsoE}=\frac{-\alpha_{R}+\sqrt{\alpha_{R}^{2}+4\beta_{R}\left(\alpha_{B}D_{B}+\alpha_{n}D_{n}+\alpha_{\gamma }D_{\gamma }+G_{B}\beta_{B}D_{B}^{2}+G_{n}\beta_{n}D_{n}^{2}+G_{\gamma }\beta_{\gamma }D_{\gamma }^{2}+MQ\right)}}{2\beta_{R}G_{R}}$$

with

(9) $$\begin{array}{ccc} MQ & = & 2G_{Bn}\sqrt{\beta_{B}\beta_{n}}D_{B}D_{n}+2G_{B\gamma }\sqrt{\beta_{B}\beta_{\gamma }}D_{B}D_{\gamma }\\ & & +2G_{\gamma n}\sqrt{\beta_{\gamma }\beta_{n}}D_{\gamma }D_{n}. \end{array}$$

### 2.4 Treatment planning

The simulation of the treatment planning was carried out using the IT_STARTS treatment planning system (TPS). This software was created in Pavia in the frame of the Istituto Nazionale Fisica Nucleare (INFN‐Italy) homonymous project, in collaboration with the Computational Physics and Radiation Biophysics Division group at the Comisión Nacional de Energía Atómica (CNEA‐Buenos Aires, Argentina). This tool is a system that inputs the medical images of a patient and the regions of interest (ROIs), producing an input file for the Monte Carlo calculation of the dosimetry, including the neutron source and patient positioning.

IT_STARTS allows the computation of photon‐equivalent dose by integrating the radiobiological data, the fit, the radiobiological parameters and the models. As the output, it delivers different Figures of Merit such as dose volume histograms (DVH), Isodose curves, tumor control probability (TCP), normal tissue complication probability (NTCP) and others. IT_STARTS is written in Python, and the TPS code itself, will be soon made public through appropriate software‐sharing methods to extend its use to different BNCT users.

### 2.5 Clinical case

**FIGURE 4:**
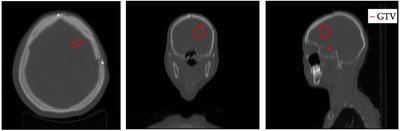
Medical images of the patient (axial, coronal, sagittal) with the GTV ROI marked by the red polygon. GTV, gross tumor volume; ROI, region of interest.

As a relevant GBM patient example, the dosimetry calculation was obtained in a clinical case treated with BNCT with the epithermal neutron source of the Tsing‐Hua Open‐Pool Reactor(THOR) at National Tsing‐Hua University.^24^ The idea to use a real patient instead of a general phantom is to work with a clinically relevant scenario, where the ROIs, the patient positioning, the boron concentration measured, the criteria for dose prescription are those applied in the real application. The gross tumor volume (GTV) ROI is shown in Figure 4 overlaid on the CT scan of the GBM patient.

The patient received a continuous infusion of L‐BPA for a total infused dose of 450 mg/kg body weight, divided into 180 mg/kg per hour for 2 h before BNCT, and 90 mg/kg per hour for drug concentration maintenance during the neutron irradiation process. Blood boron concentration was evaluated through inductively coupled plasma with atomic emission spectroscopy (ICP‐AES) during the first and second hour of L‐BPA infusion, and after the neutron irradiation. The boron concentration during the second hour is also the boron concentration when the neutron irradiation begins.^25^ The tumor‐to‐normal tissue boron concentration was calculated from the measured values in blood and using the results of a previous positron emission tomography (PET) scan. Before the treatment, the patient underwent PET with 4‐borono‐2‐18F‐fluoro‐phenylalanine (FBPA) at the Department of Nuclear Medicine of the Taiwan Veteran General Hospital, to evaluate the distribution of L‐(4‐10‐borophenyl) alanine (L‐BPA). The tumor‐to‐normal tissue ratio (T/N ratio) and the tumor‐to‐blood ratio (T/B ratio) were calculated employing the maximum standardized uptake value (SUV) of the brain and tumor. In this case the T/B ratio was 3.05 and the boron concentration was 40 ppm in tumor. Normal‐brain to‐blood ratio was 1.05 (13.6 ppm in brain).

The beam configuration was set to maximize the dose to the tumor, with the dose to the normal brain prescribed so that 50% of the volume received no more than 2.5 Gy (RBE).

In Taiwan, dosimetry was calculated using fixed RBE values obtained by Coderre et al.^12^ derived in vivo ‐ in vitro using a rat model of GSM and for normal brain values taken from.^12^ IT_START software was used to reproduce the same treatment planning using the epithermal neutron beam of the INFN RFQ accelerator described in ref. [4] as the neutron source. Once the geometry of the patient was obtained, the treatment room was simulated with the patient in the same position as the real treatment in Taiwan, considering that CPE conditions are satisfied^26^ in the healthy brain and tumor. KERMA rates were calculated with MCNP's F4‐tally type using brain KERMA factors from ICRU 44.^27^ Four absorbed dose rate matrices are the output of the simulation, one for each BNCT dose component.

## 3 RESULTS

### 3.1 Photon isoeffective dose model for GBM

#### 3.1.1 The photon reference radiation

**FIGURE 5:**
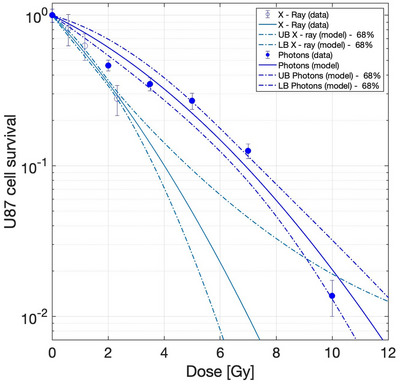
Experimental data and fit of U87 cell survival as a function of the dose of photons emitted by 60‐Co (line) and x‐rays (dash line). The dash‐dot lines represent the confidence intervals of the parameters obtained from the fit of the two curves.

To assess the impact of using different types of reference radiation, radiobiological parameters were obtained by considering the photons emitted by cobalt 60 and x‐rays. Figure 5 shows the two curves. The error bars of the experimental data represent the uncertainty in the counting of the colonies (comparable to the marker size in the figure). Table 2 reports the parameters obtained from the fitting using the modified linear quadratic (MLQ) model (Equation ( 1)).

**TABLE 2:** Values of the radiobiological parameters alpha and beta obtained by fitting the cell survival curves of the two reference radiations.

	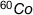	X‐ray
$\alpha_{\gamma }\;\left[Gy^{-1}\right]$	0.21±0.08	0.46±0.09
$\beta_{\gamma }\;\left[Gy^{-2}\right]$	0.02±0.01	0.03±0.04

*Note*: The associated errors are those of the parameters with a confidence interval of 68%.

Figure 5 shows that the two curves are significantly different. A measure to quantify this difference is the RBE for a specific endpoint of x‐rays compared to gamma. The RBE for a survival fraction of 0.01 is 1.5. The fact that the biological effectiveness of x‐rays is higher than that of gamma rays has been observed before, see for example.^19^ This result impacts on the RBE and CBE factors for BNCT for a specific endpoint, and, in turn, also on the photon isoeffective dose.

#### 3.1.2 Beam‐only and BPA‐BNCT radiobiological parameters

**FIGURE 6:**
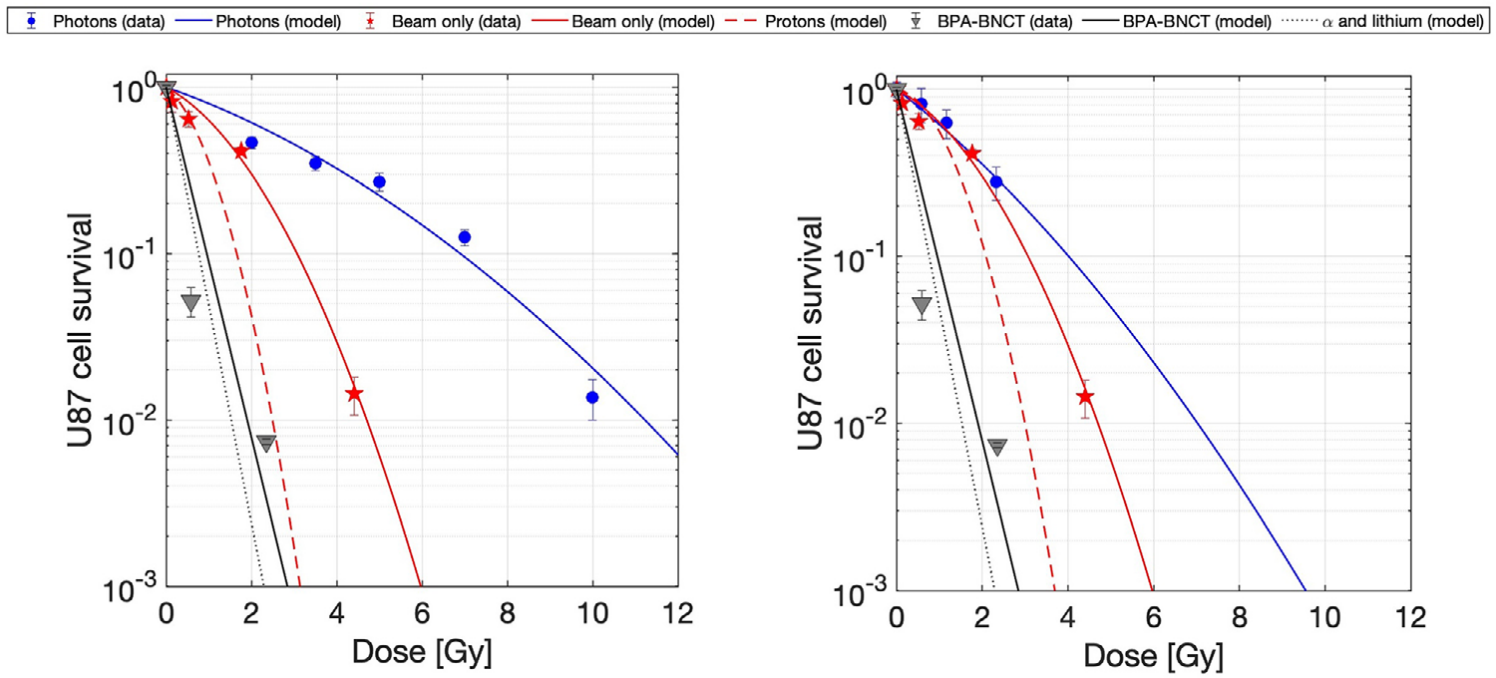
Survival curves of U87 cells as a function of the absorbed dose. Left: using 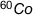 as the reference radiation. Right: using x‐ray as the reference radiation. The blue dots and line represent the data from the reference radiation and the fit, respectively. The red stars and the solid curve are the experimental data and the fit for the beam‐only irradiation, respectively. The red dashed curve is the isolated contribution of protons (i.e. subtracting the effect of photons). The solid black curve shows the fit for the BPA‐BNCT experimental points (black triangles). The dotted black line is the model considering only the boron component (i.e., subtracting photon, proton and 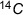 contributions). BNCT, Boron neutron capture therapy; BPA, Boronophenylalanine.

The beam‐only and BPA‐BNCT curves were simultaneously fitted with the MLQ model (Equation (2)) considering the x‐rays or the Co‐60 gamma radiation as the reference. The fitted curves are reported in Figure 6. The horizontal error bars for the doses are dominated by the uncertainty in boron concentration measurements. The fit procedure was error‐weighted.

Table 3 shows the radiobiological parameters obtained from the fit of the experimental data of cell survival as a function of dose for beam‐only and BPA‐BNCT in the U87 cell line when the reference radiation comes from the Co‐60 source and from the x‐ray irradiator, respectively.

**TABLE 3:** Radiobiological parameters for GBM, obtained from the fit of the cell survival curves as a function of dose for beam‐only and BPA‐BNCT irradiation (boron component), using as radiation reference and X‐Ray.

Radiobiological parameters for GBM				
	Photons from 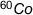		Photons from x ‐ ray	
	Neutrons	Boron	Neutrons	Boron
$\alpha \;[Gy^{-1}]$	0.5 ± 0.9	3.0 ± 0.6	0.1 ± 0.9	3.0 ± 0.6
$\beta \;[Gy^{-2}]$	0.5 ± 0.6	0	0.5 ± 0.6	0

*Note*: Uncertainties correspond to a 68% confidence level.

Abbreviations: BNCT, Boron neutron capture therapy; BPA, Boronophenylalanine; GBM, glioblastoma multiforme.

Given the different radiobiological parameters derived from the curves, the RBE and CBE factors vary accordingly. Table 4 lists the values of RBE and CBE factors calculated for 1% of survival with the curves that have the isolated $\text{proton}{+}^{14}C$ and $\alpha {+}^{7}Li$ contributions (dashed red line and dotted black lines in Figure 6 respectively). Moreover, it reports the values taken from,^12^ typically used in BNCT clinical applications.

**TABLE 4:** ${\text{RBE}}_{1\%}$ and ${\text{CBE}}_{1\%}$ values, calculated considering the two reference radiations and the one used in the clinic.

	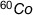	X‐ray	Clinic
**RBE**	4.8±0.7	2.5±1.4	3.2
**CBE**	7.5±0.9	4.7±1.5	3.8

*Note*: The errors associated is the propagation of the uncertainties of the fit parameters.

Abbreviations: RBE, relative biological effectiveness; CBE, compound biological effectiveness.

The results clearly show that the reference radiation used has an appreciable impact on the RBE/CBE factors that relate to the biological effectiveness of other radiation compared to photons, see Table 5. Furthermore, compared with those used in the BNCT clinic obtained for GSM with x‐ray photons, it can be seen that the results obtained in this work are significantly different. The choice of the reference radiation plays thus a significant role in the translation of BNCT dose in photon‐equivalent units. Considering the motivation that the photon isoeffective dose is calculated to predict a clinical outcome based on the experience gained in conventional radiotherapy, we used Co‐60 as the reference radiation for the in‐patient dosimetry.

**TABLE 5:** Percentage differences in RBE and CBE values between the two reference radiations (Cobalt and x‐ray) and between the two photon sources and the values used in the clinics.

Relative differences [%]			
	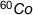 versus X‐ray	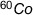 versus Clinic	X‐ray versus Clinic
**RBE**	44	29	29
**CBE**	37	49	19

Abbreviations: RBE, relative biological effectiveness; CBE, compound biological effectiveness.

Thus, the overall set of radiobiological parameters for the photon isoeffective dose model optimized for GBM are summarized in Table 6. These are the parameters that must be inserted in Equation 8.

**TABLE 6:** Radiobiological parameters for GBM.

Radiobiological parameters for GBM			
	Photon	Neutron	Boron
$\alpha \;[Gy^{-1}]$	0.21 ± 0.08	0.5 ± 0.9	3.0 ± 0.6
$\beta \;[Gy^{-2}]$	0.02 ± 0.01	0.5 ± 0.6	0

*Note*: Uncertainties correspond to a 68% confidence level.

Abbreviation: GBM, glioblastoma multiforme.

### 3.2 In‐patient dosimetry

The dose prescription to healthy brain led to an irradiation time of 33.3 min using the INFN neutron beam. With this irradiation time, the photon isoeffective dose in the tumor was calculated with radiobiological parameters derived from U87 experiments and the one from GSM. This comparison is meant to highlight the impact of using parameters coming from different tumor models in the calculation of photon‐equivalent dose in patients.

**FIGURE 7:**
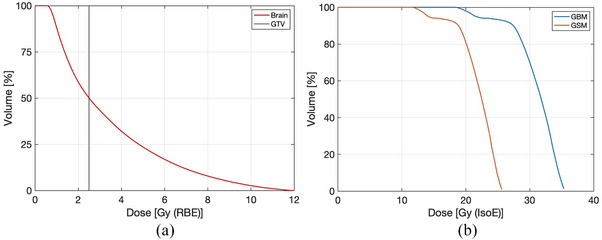
(a) DVH of the healthy brain; the black vertical line highlights the prescription limit of 2.5Gy(RBE) or less to 50% of the brain volume. (b) DVH for the tumor (GTV) calculated using the photon isoeffective dose model with parameters derived from glioblastoma (Blue) and from GSM (Red). DVH, dose‐volume histogram; GSM, gliosarcoma; GTV, gross tumor volume.

The dose in normal brain was obtained using the radiobiological data from,^12^ to respect the dose prescription adopted in the original treatment. Figure 7 shows the DVH of the healthy brain in the left panel. The black vertical line represents the RBE limiting dose for the organ at risk set by Taiwan protocol. The right panel depicts the DVH of the Gross Tumor Volume, GTV, obtained by calculating the absorbed dose using the Photon Isoeffective Dose Model. The red curve is obtained with the radiobological parameters of the rat‐gliosarcoma experiments and the blue one is obtained with the U87 experiments described above.

Table 7 reports the maximum, mean and minimum doses for the RBE‐weighted model and the photon isoeffective model. The two dose models were fed with the parameters derived from GMB and GSM experiments.

**TABLE 7:** Maximum, mean and minimum dose values for the two models considered, each fed with parameters from two different biological systems (GBM and GSM).

	Gross tumor volume			
	Photon isoeffective model [Gy (IsoE)]		RBE‐weighted model [Gy (RBE)]	
	GSM	GBM	GSM	GBM
$D_{max}$	25.5	35.2	41.1	76.2
$D_{mean}$	22.5	31.8	34.6	63.4
$D_{min}$	13.0	20.0	16.4	29.5

Abbreviations: RBE, Relative Biological Effectiveness; GBM, glioblastoma multiforme; GSM, gliosarcoma.

Table 8 shows the percentage differences of the dose values for the RBE‐weighted and isoeffective dose model, feeding them with parameters derived from GBM and GSM.

**TABLE 8:** Percentage difference due to the set of radiobiological data used (GSM vs. GBM) in maximum, average and minimum dose values for the RBE‐weighted model (second column) and for the isoeffective model (third column).

	Photon Isoeffective Model	RBE ‐ weighted Model
	GBM ‐ GSM	GBM ‐ GSM
Max	28 %	46 %
Mean	29 %	45 %
Min	35 %	44 %

Abbreviations: RBE, relative biological effectiveness; GBM, glioblastoma multiforme; GSM, gliosarcoma.

## 4 DISCUSSION

This study tackles the necessity of implementing an appropriate model for photon‐equivalent dosimetry in BNCT for GBM. A robust model is crucial for explaining retrospective clinical outcomes in view of their dosimetry and predicting the therapeutic effectiveness of treatment planning for future clinical trials. For the first time, the influence of the selected photon reference radiation on the calculation of the radiobiological parameters needed to feed the dose model has been measured and proved to be quantitatively relevant. It should be noted that cell survival experimental data depicted in Figure 6 show a particular trend at a low dose region, which would merit further experiments to verify whether it represents a specific characteristic of the cell line studied.

Two standard photon sources for radiobiological experiments yielded RBE/CBE factors for the neutron and boron components exhibiting differences up to 44%. Therefore, when calculating a photon equivalent dose using x‐rays, the resulting BNCT dose does not accurately reflect the effect achieved by clinical photons. In this work, we started experiments with the x‐ray irradiator available at the laboratory because this is often the typical choice. However, we have then shifted to a more representative photon source and it has been important to highlight the significance of selecting a proper reference when it is necessary to compare BNCT effects to the ones of clinical radiotherapy (energies of the order of MeV).

Having a set of radiobiological parameters derived from a GBM model (U87 human cell line), the dose distribution was calculated in a real clinical case comparing the results with those obtained with the existing parameters derived from experiments on the rat 9L GSM cell line. The difference between the dose values obtained is significant, as reported in Table 8. This shows that consistent radiobiological data should be available for each tumor. The same difference was found within the standard model considering fixed values of RBE and CBE calculated with the two radiobiological datasets, see Table 8. Albeit the availability of radiobiological data concerning a human GBM is already a significant improvement in the photon‐equivalent dosimetry, the irradiation of additional cell lines of GSM would be certainly contributory to assess the clinical significance of the selection of proper pre‐clinical models. A further step would be the irradiation of small animals with GBM to measure the TCP as a function of the dose, and to calculate photon‐equivalent dose based on in vivo effects.

The dose calculations in patients are performed assuming uniform boron distribution at the tissue and cell levels. Currently, clinical treatment planning does not consider that the tumor is made up of different cell populations that may absorb different boron concentration. To our knowledge, no existing model used in the clinical application considers this lack of uniformity. Photon isoeffective dose model could easily take into account different boron concentrations at the tissue level, when the experimental information is available. The fact that boron may be non‐uniform at the sub‐cellular level (i.e. nucleus vs cytoplasm), however, requires an extension of the model with a proper formalism which differentiate the dose deposition in the cells according to the micro‐distribution of boron.

Regarding the difference between the standard and the photon isoeffective dose model, we have confirmed what has been extensively shown in other works, that is, tumor doses are overestimated when calculated with RBE‐weighted model.^13^, ^28^, ^29^, ^30^ The RBE‐weighted tumor doses are significantly higher than the photon isoeffective dose values, especially as the total absorbed tumor dose increases. The overestimation of dose in tumor has a consequence for clinical BNCT because it influences the capacity of assessing a proper dose‐effect relation, preventing the prediction of a clinical outcome based on the dose distribution calculated by the treatment planning. If the dose were that high, it would not be necessary to limit the dose to normal tissues and prescribe the dose to the tumor as in conventional radiotherapy. However, the BNCT clinical outcomes do not correspond to such high doses, in light of photon therapy experience. This suggests that the dose calculation model that are typically used to convert mixed‐field radiation dose into photon‐equivalent units is not correct. On the other hand, the photon isoeffective dose values calculated in this work, are much more consistent with those delivered by single fraction photon RT for the treatment of brain tumors.^31^

Benevicious et al. analyzed GBM patients treated with stereotactic radiosurgery.^32^ They showed that with a maximum single‐fraction dose of 30.45 +/‐ 7.46 Gy and a margin dose of 15.30 +/‐ 3.19 Gy, the local control (complete response, partial response and stable disease) was 68%. On the other hand, BNCT GBM patients treated in Finland^33^ showed a local control of 60% at the same follow‐up time, with doses in the range 16‐59 Gy‐Eq, using the RBE‐weighted model. The fact that RBE‐weighted dose values are significantly higher than those in [32] for a similar clinical outcome reinforces the idea that the traditional model overestimates the tumor dose. In the patient that we analyzed, RBE‐weighted doses are similar to what was calculated by Kankaanranta et al. On the other hand, the photon isoeffective dose is consistent with photon therapy data in [32]. These findings strongly suggest that the isoeffective dose model is an adequate tool to calculate photon‐equivalent doses. Therefore, the possibility of using the TCP derived from clinical experience with photons to prospectively assess the therapeutic potential of BNCT treatment for GBM is now open.

## 5 CONCLUSIONS

Improvements in the capacity to calculate a reliable photon equivalent dosimetry for BNCT are very urgent to prescribe a proper dose and to predict the clinical outcome from a simulated treatment plan. Photon therapy is the typical reference because there is long experience and a very large amount of data on patients. Usually, in radiobiological experiments, x‐rays are used as the reference. We showed here that radiobiological parameters obtained with different photon sources are very different. This discrepancy is reflected in the treatment planning of a patient, as the radiobiological parameters obtained from the fit, are used to feed the photon isoeffective model. To prescribe the dose for controlling the tumor while sparing the surrounding healthy tissue, it is necessary know the dose‐effect relationship. It is thus important to choose the most suitable radiobiological parameters to translate BNCT dose into photon units.

To understand the impact of the radiobiological model used in the treatment plan of a patient treated with BNCT, a clinical case of GBM was taken as a relevant example. Today, clinical BNCT dosimetry for GBM is based on RBE factors derived from in vivo/in vitro experiments using a model of rat 9L GSM. Radiobiological data on this tumor are available in the literature, however, data on BNCT of GBM were still missing. A set of new data on GBM was produced in Pavia, and used in the dosimetric models. The differences in dose values calculated demonstrate that it is very important to select a correct dosimetric model and consistent radiobiological data. Following what already obtained for head and neck cancer^28^ and melanoma^13^ the dosimetric models will be validated in retrospective analysis using radiobiological figures of merit as the TCP. A robust dosimetry in photon‐equivalent unit is the one which predicts a number of controlled tumor similar to what observed in clinical trials. To this end, a TCP model for GBM and including non‐homogeneous dose distribution is being developed. Furthermore, more cell lines of GBM and in vivo models will be employed to deepen the knowledge and improve the description of the effect of BNCT on GBM as a function of its mixed‐field dose. Finally, future work will assess the impact of the micro‐distribution of boron in tumor cells including this information in the photon isoeffective dose model.

## CONFLICT OF INTEREST STATEMENT

The authors declare no conflicts of interest.
